# [Corrigendum] Tripartite motif‑containing 11 regulates the proliferation and apoptosis of breast cancer cells

**DOI:** 10.3892/or.2024.8838

**Published:** 2024-11-06

**Authors:** Xianping Dai, Feng Geng, Mengshun Li, Ming Liu

Oncol Rep 41: 2567–2574, 2019; DOI: 10.3892/or.2019.7015

Subsequently to the publication of the above paper, an interested reader drew to the authors' attention that, concerning the flow cytometric plots shown in Fig. 5A and B on p. 2572, each figure part contained a pair of duplicated data panels; specifically, the panels depicting the ‘NC/5-FU’ and the ‘shTRIM11/Gemcitabine’ experiments in Fig 5A (MCF-7 cells), and the ‘NC/Paclitaxel’ and ‘shTRIM11/Adriamycin’ experiments in Fig. 5B (MDA-MB-231 cells), were apparently identical.

The authors were able to re-examine their original data files, and realize that this figure was inadverently assembled incorrectly. The revised version of Fig. 5, now showing the correct data for the ‘shTRIM11/Gemcitabine’ experiment in Fig 5A and the ‘NC/Paclitaxel’ experiment in Fig. 5B, is shown on the next page. Note that the revisions made to this figure do not affect the overall conclusions reported in the paper. The authors are grateful to the Editor of *Oncology Reports* for allowing them the opportunity to publish this Corrigendum, and apologize to the readership for any inconvenience caused.

## Figures and Tables

**Figure 5.**
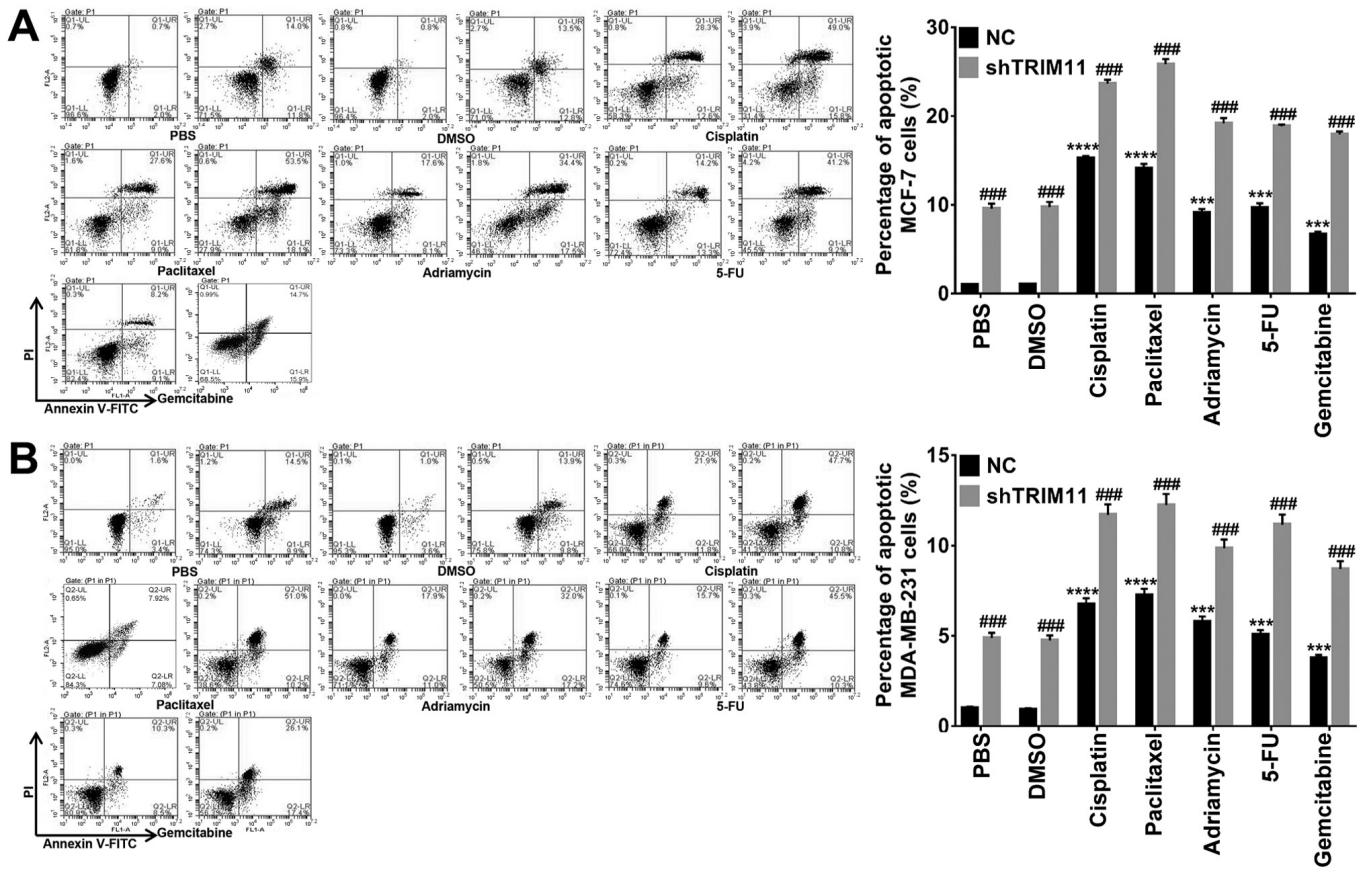
Downregulation of TRIM11 enhances the effects of chemotherapeutic drugs on the apoptosis of (A) MCF-7 and (B) MDA-MB-231 cells. Following infection with the shTRIM11 lentivirus, (A) MCF-7 and (B) MDA-MB-231 cells were individually treated with five chemotherapeutic drugs (5 µg/ml). After 24 h, the cells were collected to determine their apoptosis rates by flow cytometric analysis. PBS and DMSO were used as vehicle controls. Data are presented as the mean ± standard deviation. ***P<0.001, ****P<0.0001, compared with vehicle controls; ^###^P<0.001, compared with NC. TRIM11, tripartite motif-containing 11; NC, negative control; sh, short hairpin RNA; DMSO, dimethyl sulfoxide; 5-FU, fluorouracil..

